# Complications after oesophagectomy with possible contribution of neoadjuvant therapy including an EGFR-antibody to a fatal outcome

**DOI:** 10.1186/1477-7819-5-114

**Published:** 2007-10-11

**Authors:** Michael Knauer, Anton Haid, Karlheinz Ammann, Alois Lang, Felix Offner, Martina Türtscher, Peter Cerkl, Etienne Wenzl

**Affiliations:** 1 Department of General and Thoracic Surgery, General Hospital of Feldkirch, Academic Teaching Hospital, Feldkirch, Austria; 2 University of Human Sciences, Principality of Liechtenstein, General Hospital of Feldkirch, Academic Teaching Hospital, Feldkirch, Austria; 3 Department of Internal Medicine, General Hospital of Feldkirch, Academic Teaching Hospital, Feldkirch, Austria; 4 Department of Pathology, General Hospital of Feldkirch, Academic Teaching Hospital, Feldkirch, Austria; 5 Department of Pulmonology, General Hospital of Feldkirch, Academic Teaching Hospital, Feldkirch, Austria

## Abstract

**Background:**

Different molecular therapies like the EGFR-inhibiting antibody cetuximab have come into clinical practice. Cetuximab is EMEA-approved for metastatic colorectal cancer and advanced squamous-cell head and neck cancer. Administration is said to be safe and well tolerated with common, usually mild dermatologic side effects.

**Case presentation:**

We present the case of a patient with fatal complications after oesophagectomy and neoadjuvant chemotherapy including cetuximab for squamous-cell esophageal cancer. A transthoracic *en-bloc *oesophagectomy was performed. Few days later the patient died due to gas exchange dysfunction and circulation instability after a previously unseen combination of drain-erosion of the stomach with subsequent pleurisy and air leak of the left main bronchus.

**Conclusion:**

So far we have never observed this fatal combination of drain erosion of the stomach with fibrinous pleurisy and unmanageable progressive tracheal defect before. The role of cetuximab in the multifactorial aetiology of damages of stomach and trachea after oesophagectomy remains unclear since we are not able to link the complication directly to cetuximab or definitely exclude it as a sole surgical complication. Clinicians should be aware of the possibility of fatal side effects and careful recording of all complications is necessary in ongoing and planned studies to obtain more evidence about safety and tolerance of targeted therapies.

## Background

Oesophageal cancer represents the sixth leading cause of cancer-related death in the world. Despite recent advances in surgical critical care medicine and combined modality therapies 5-year overall survival rates (10–14%) are unsatisfactorily low [1]. The only curative therapy in localized cancer is provided by radical surgery. However, more than 50% of all patients are diagnosed with inoperable or metastatic disease [2]. Next to radical surgery compared with chemoradiotherapy alone [3], neoadjuvant chemotherapy approaches have been studied with a pathologic complete response rate (pCR) of up to 24% [4]. Although some authors state that still no standard recommendation can be given for a multimodality therapy outside clinical trials [5], randomised trials exist showing survival benefits after neoadjuvant chemotherapy and therefore neoadjuvant chemotherapy is considered part of standard practice in many institutions[6].

In the last decade different molecular therapies have changed the field of research, trying to inhibit or modulate targets of signal transduction pathways. One of those that made it into clinical practice is the epidermal growth-factor receptor (EGFR) inhibiting chimeric antibody cetuximab (Erbitux, Merck Pharma Gmbh, Darmstadt, D). This monoclonal antibody blocks EGF and TGF-α binding to the extracellular domain of EGFR, which results in cell-growth inhibition, induction of apoptosis and decreased production of EGF [7].

Cetuximab is EMEA-approved for second line treatment of EGFR-expressing metastatic colorectal cancer refractory to irinotecan-based chemotherapy and locally advanced squamous-cell head and neck cancer with concomitant radiotherapy. Many solid tumors including esophageal cancer overexpress EGFR, predicting poor survival, poor response to therapy as well as higher probability for disease progression and resistance to therapy [8-10]. This makes cetuximab a promising anticancer agent for different neoplasms, but so far no clinical trials have been reported in esophageal cancer patients. Ongoing trials include two studies in metastatic esophageal cancer (South-west Oncology Group trial and Memorial Sloan-Kettering Cancer Center study) and one preoperative phase II trial with cisplatin, irinotecan, cetuximab and radiation at Dana-Farber Cancer Institute [11].

Generally EGFR-antibodies (e.g. cetuximab, matuzumab, panitumumab) or EGFR tyrosine kinase inhibitors (e.g. gefitinib, erlotinib) are said to be safe and well tolerated without systemic side-effects of chemotherapy. Common dermatologic side effects of cetuximab in a considerable number of patients are acneiform eruptions, xerosis, eczema, fissures, teleangiectasia, hyperpigmentation, hair changes and paronychia [12]. More severe adverse reactions are grade 3 to 4 allergic reactions and severe dyspnea.

In this report we present a case report with fatal postoperative complications after neoadjuvant chemotherapy including cetuximab for squamous-cell esophageal cancer and discussion of the literature.

## Case presentation

A 52-year old man was diagnosed with squamous-cell esophageal cancer of the lower third. Pretherapeutical investigations included endoscopical biopsy, CT scan, endosonography and mediastinoscopy with lymph-node biopsy. These investigations showed a locally advanced stage T4N1 cancer.

**Figure 1:**
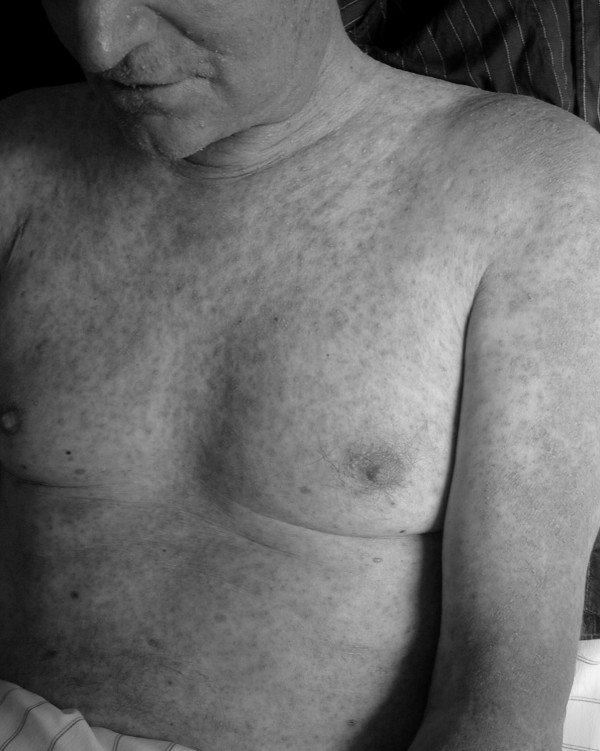
Severe acute generalized exanthematic pustulosis (AGEP) five weeks after first cycle of cetuximab therapy.

The patient was scheduled for two cycles of neoadjuvant radiochemotherapy with cisplatin 100 mg per m^2 ^and 5-FU 1000 mg per m^2^. After the first chemotherapy cycle the patient developed grade 3 mucositis and esophagitis combined with an infection of the port-a-cath system, which had to be removed. This intense toxicity gave us reason to search for a dihydropyrimidin-dehydrogenase-deficiency. The result of the genetic testing was negative. Because of the toxic esophagitis and mucositis the patient refused to undergo the planned radiotherapy. From the second cycle continous 5-FU was replaced by oral capecitabine because of the port-a-cath infection and cetuximab was added as an alternative to radiotherapy after informed consent in a compassionate use setting. The EGFR-testing had shown a strong overexpression in all tumor cells. The treatment consisted of an intravenous standard loading dose of 400 mg per m^2 ^after administration of diphenhydramine and ranitidine and continued with 250 mg per m^2 ^once weekly for four weeks. After five weeks the patient developed disseminated pustules with generalized deeply infiltrated erythematous plaques highly indicative for a severe acute generalized exanthematic pustulosis (AGEP) as shown in figure 1. These symptoms diminished after four days of dexamethasone, cefuroxime, silver sulfadiazine cream and diphenhydramine therapy. Because of this severe adverse effect cetuximab was stopped and a restaging CT scan was performed.

**Figure 2:**
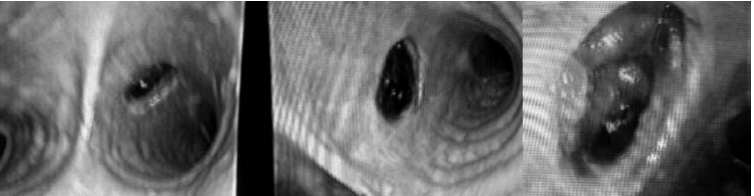
Unmanageable increase of tracheal damage within four days. Left picture with a small 0.5 cm defect, in center defect increasing after 2 days and the rightmost picture shows a 2 cm defect of the left main bronchus short before death of the patient.

Reevaluation showed nearly no remission of the tumor as well as stable disease in the suspect lymph nodes. Because of lack of response and the intense toxicity the patient wanted to stop the neoadjuvant therapy and proceeded to transthoracic *en bloc *oesophagectomy five weeks after the last cetuximab application. Replacement of the esophagus was performed with an orthotopic gastric tube and cervical esophago-gastrostomy. On the third postoperative day a leakage from the right thoracic drain was observed and the following immediate revision operation showed an erosion of the stomach, which was most likely caused by a thoracic drain positioned in the vicinity of the gastric tube. The anastomosis 3 cm above the defect was completely intact with macroscopic sufficient blood circulation. Because of an additional fibrinous pleurisy we had to conduct a disconnection esophagostomy, catheter-gastrostomy and -jejunostomy for early enteral feeding. After four days the patient developed an airleak of the left main bronchus just below the tracheal bifurcation. At the time of the first bronchoscopy we found a 5 mm ulcer where the tracheal tube cuff was located during the operation until extubation on the first postoperative day. Within four days the defect's size increased by fourfold as shown in figure 2 and a tracheal stent had to be implanted. This stent successfully closed the leak for several hours until a second leakage above the first was observed which was covered by the cuff of the tracheal tube. Neither the stent nor the cuff could successfully reduce the airleak and the situation demanded a right-sided intubation. Three days later the patient died due to refractory gas exchange dysfunction and circulation instability.

### Surgical treatment after neoadjuvant therapy

The patient's squamous-cell esophageal cancer of the lower third (35 – 38 cm from incisor teeth) was treated by *en bloc *transthoracic oesophagectomy with paraesophageal, paratracheal, aorto-pulmonary and paracardial lymphadenectomy including lymph nodes of the splenic, hepatic and celiac arteries followed by cervical esophagogastrostomy under general anesthesia with separate intubation. The operation time was 4.5 hours and the blood loss approximately 600 ml. The reoperation consisted of a disconnection esophagostomy, catheter-gastrostomy and -jejunostomy. The tracheal leakage was treated by a 12 French Dumont silicone stent.

### Tumor

Histologic tumor studies were done by haematology and eosin staining and EGFR-overexpression was measured by EGFR Pharm Dx kit K 1494 (Dako Cytomation).

### Surgical specimen

The oesophageal tumour was staged as ypT3 N1(9/25) MX R0 on final pathology. After reoperation the histologic examination of the resected specimen revealed extensive ischemic necrosis of the gastric mucosa with erosions and microabscesses, oedematous submucosa and the muscular and serosal layer next to the defect in good order.

### Autopsy

**Figure 3:**
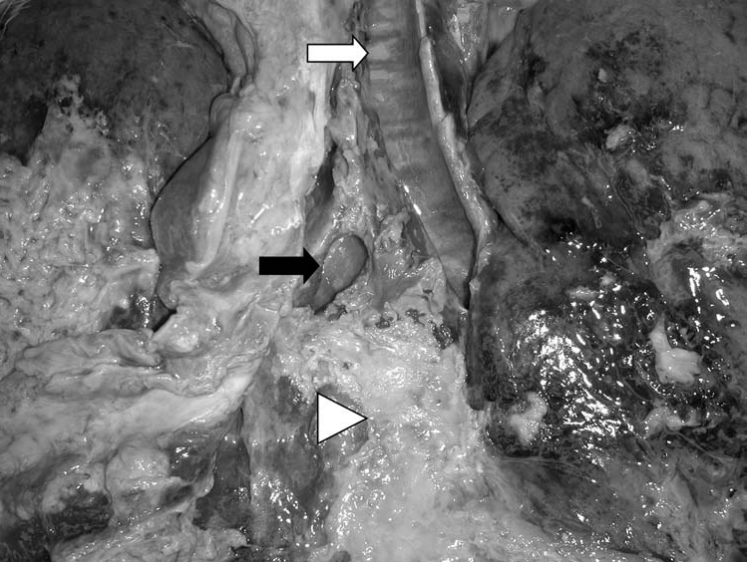
Autopsy results: inflammatory trachea (white arrow), tracheal leak in left main bronchus (black arrow), fibrinous pleurisy (white triangle).

The autopsy showed massive fibrinous pleurisy, mediastinitis, left-sided bronchitis and bronchopneumonia in addition to the 2 cm tracheal defect of the membranous part of the left main bronchus. Histological complete tracheal wall destruction was observed with fibrin, necrotic areas and focal formation of granulation tissue without evidence of residual tumour (figure 3).

## Discussion

So far we have never observed this fatal combination of drain erosion of the stomach with fibrinous pleurisy and unmanageable progressive tracheal defect. Tracheal fistulae with airleaks are rare but life threatening complications after esophageal resections one to thirty days after oesophagectomy. The reported incidence of tracheal lesions is about 4 percent and about a third of the patients dies during the postoperative course, mostly because of unhealed lesions at the bifurcation or in the left main stem bronchus [13]. Ischemia after peritracheal dissection was the pathologic explanation in about half of the cases.

Preoperative radiochemotherapy predisposed to this complication significantly and also in our patient neoadjuvant chemotherapy including cetuximab was administered. In the interdisciplinary tumorboard review a multifactorial event was discussed with cetuximab possibly playing a role. Other predisposing factors such as tracheal ischemia after extended mediastinal lymphadenectomy and damage of the membranous part after perforation of the stomach with subsequent mediastinitis have certainly contributed to the lethal outcome.

Since the reassessment of EGFR-expression in the gastric and tracheal tissue was not possible one can only speculate about a possible contribution of cetuximab in addition to surgical complications after extensive lymphadenectomy.

Yanaka *et al*., described the important role of EGF in restitution of gastric mucosae in guinea pigs, that was significantly inhibited by an anti-EGFR antibody [14]. Tarnawski *et al*., demonstrated that EGF inhibits acid secretion, exerts a trophic effect on gastroduodenal mucosa, protects gastric mucosa against injury, mediates mucosal adaptation and accelerates gastroduodenal ulcer healing by stimulating cell migration and proliferation [15]. Regarding the antivascular endothelial growth factor antibody bevacicumab, several patients with severe bowel complications such as bowel perforation have been described [16] and several tracheo-esophageal fistula have been reported which prompted a warning from the manufacturer. No data exist with regard to neoadjuvant cetuximab therapy, where only small aphthous ulcers of the oral mucosa have been reported to date [17].

Considering the bronchial system Barrow *et al*., hypothesised that EGFR ligands and other growth factors mediate bronchial epithelial repair in sheep [18]. White *et al*., demonstrated in human airway epithelial cells that EGF induced epithelial repair via cell migration after mechanical injury [19]. Puddicombe *et al*., showed that EGF-promoted wound closure in airway epithelial cells was retarded by a selective EGFR tyrosine kinase inhibitor (AG 1478) [20]. These preclinical data and the fact that despite of a surgical complication we have never observed such a combination of drain erosion of the complete stomach wall and a non-manageable progressive tracheal defect rise the question if a correlation between the neoadjuvant cetuximab therapy in our patient and impaired EGFR-mediated repair mechanisms in gastric and airway epithelial cells exists. Unfortunately we could neither prove a causal influence of cetuximab on the outcome nor definitely exclude a sole surgical complication, therefore we tried to calculate the possibility of an adverse drug reaction as shown below. In a phase II study adding cetuximab to neoadjuvant chemoradiotherapy in advanced oesophageal cancer no such severe postoperative complications were observed in 15 operated patients although early results suggested a lower complete response rate and higher overall toxicity. One of the seventeen study patients died after respiratory failure and sepsis, chemotherapy dose attenuation was required in 10 patients and one patient was removed from the study due to prolonged diarrhoea [11].

### Assessment of the adverse drug reaction

Adverse drug reactions are suspected to be the 4^th ^to 6^th ^leading cause of death in the US causing 106.000 fatalities per year [21] although the meta-analayis has been heavily criticized by others [22]. Cullen *et al*., have demonstrated that voluntary reporting identifies only about 6% of ADRs in the clinical routine and improvement is essential [23]. Linking these fatal complications after oesophagectomy to an ADR after cetuximab is not that simple and this remains the important limitation to this report. The common dermatologic reaction of acneiform eruption confined to the face, scalp, chest and upper back areas increased to a severe acute generalized exanthematic pustulosis (figure 1), that was the reason to stop the neoadjuvant treatment and continue with surgical treatment. We tried to determine the probability of cetuximab causing the events and to classify them as an ADR using systematic criteria such as the algorithm of Naranjo and colleagues that is commonly used [24]. The total score in the Naranjo algorithm was two points, showing a "possible probability". On the other hand five of the ten questions were not applicable in our patient such as improvement of symptoms after medication discontinuation or reappearance after readministration or placebo because the events were delayed after neoadjuvant therapy and the outcome was fatal. So the clinical usefulness of this algorithm was limited and has been questioned before by other authors for the use in critical ill ICU patients [25].

The role of cetuximab in ischemic damages of the stomach and trachea after ooesophagectomy is unclear. While Bartels *et al*., found tracheal complications in 4% after 785 esophagectomies (10.3% after radiochemotherapy but none after chemotherapy alone) [13]. Kelley *et al*., found no evidence that induction therapy adversely influences the incidence of postoperative morbidity or mortality after oesophagectomy in 155 patients [26].

## Conclusion

Preclinical evidence exists for a possible correlation between EGFR-antibody therapy and impaired EGFR-mediated repair mechanisms in gastric and airway epithelial cells. The widespread, in general safe and well-tolerated use of cetuximab in metastatic colorectal and locally advanced squamous-cell head and neck cancer has to lead to further investigations in neoadjuvant settings also in other cancers such as esophageal cancer. In our patient with severe postoperative complications the etiology of this complication is likely to have been multifactorial, with cetuximab possibly playing a role. Although the suspected increase of risk for complications after oesophagectomy needs to be confirmed in prospective trials before final conclusions can be made, clinicians should be aware of the possibility of these side effects. Careful recording of all complications is necessary in ongoing and planned studies to obtain more evidence about safety and tolerance of targeted therapies since these new biologic agents may be not as safe as we have initially assumed.

## Competing interests

The author(s) declare that they have no competing interests.

## Authors' contributions

KM & HA Conception and design, writing of manuscript

WE, OF: Help in preparation of draft and editing of manuscript, data analysis and interpretation.

AK, CP, TM: Managed the case and helped in preparation of draft

KM, LA, TM: Collection and assembly of data

All authors read and approved final manuscript for publication.
